# University students’ thriving during remote and in-person instruction in the COVID-19 pandemic: longitudinal evidence from two academic years

**DOI:** 10.3389/fpsyg.2025.1638392

**Published:** 2026-01-06

**Authors:** Jannika Haase, Lysann Zander

**Affiliations:** Division of Empirical Educational Research, Institute of Education, Leibniz Universität Hannover, Hanover, Germany

**Keywords:** thriving, higher education, remote/in-person instruction, gender, college generation status

## Abstract

**Introduction:**

A substantial body of research has demonstrated the negative repercussions of the COVID-19 pandemic on university students’ mental health and well-being. Less is known about students’ thriving, defined as a specific sense of personal growth encompassing experiences of vitality and learning.

**Methods:**

Using a longitudinal dataset (*N* = 431) from a large public university in Germany, we examined how students’ thriving developed over the course of two academic years, including five time points from June/July 2020 to February 2022, during which remote instruction (T1–T3), in-person instruction (T4) and again remote instruction (T5) were carried out. To capture intraindividual change, we used two neighbor change models, a subtype of latent change score (LCS) models.

**Results:**

During the period of remote instruction, we found intraindividual decreases in students’ thriving toward T2 in winter 2020/2021. When universities had resumed in-person instruction in winter 2021/2022 (T4), we found intraindividual increases in students’ vitality and learning. Intraindividual changes in thriving toward all later time points did not differ by gender or by college generation status.

**Discussion:**

We discuss our findings against the background of the study-related stressors that students faced during remote and in-person instruction, as well as the instructional measures implemented by the respective university over the course of the pandemic.

## 1 Introduction

The COVID-19 pandemic was considered one of the most unique and unprecedented challenging periods in education in history (United Nations, 2020). During this period, university students faced a variety of study-related stressors, often arising from rapidly changing circumstances (e.g., Karnbach et al., 2024; Matos Fialho et al., 2021). Research on stress, coping, and resilience suggests that individuals can respond to stressors in both adaptive and less adaptive ways (Lazarus and Folkman, 1984; see also Carver, 1998; O’Leary and Ickovics, 1995). While some people perceive stressors as harmful or threatening and may react less adaptively (e.g., with increased stress levels), others experience stressors as challenges and respond more adaptively, for instance, by making use of individual and social coping resources (e.g., support from others) in order to show an outcome of positive value, possibly including mastery, gain, and stress-related growth (e.g., Carver, 1998; Lazarus and Folkman, 1984; see also Kleine et al., 2019). One specific form of perceived stress-related growth is thriving, defined as a positive psychological state characterized by both vitality and learning experiences (Ozcan et al., 2023; Spreitzer et al., 2005; see also Porath et al., 2012). In the present study, we consider university students’ thriving as an adaptive response to the specific study-related stressors during the first two academic years of the pandemic. We examined students’ thriving over five time points from June/July 2020 until February 2022, including periods of remote and in-person instruction. Based on research on fluctuations in thriving over moderate periods of time (e.g., Brown et al., 2021; Kleine et al., 2023), we deemed it important to investigate potential variations in students’ vitality and learning in an extraordinary study situation along with changes in university instruction. We further investigated two characteristics of students’ demographic background (i.e., gender, college generation status) as potential predictors of thriving over time.

So far, numerous longitudinal studies have investigated university students’ mental health (e.g., Höhne et al., 2024; Robinson et al., 2022) and well-being during the pandemic (e.g., Lemyre et al., 2023). However, to our knowledge, while cross-sectional studies in higher education during the pandemic (e.g., Sahin and Tuna, 2022; Warnock et al., 2024) have identified negative associations between anxiety or negative appraisals and thriving, and longitudinal studies in pre-pandemic occupational contexts exist (e.g., Augustus et al., 2024; Kleine et al., 2023), there is still a paucity of longitudinal research on thriving among university students during the pandemic. While, to our knowledge, the only longitudinal study in higher education has examined students’ thriving over a short 9-day period (Hua et al., 2022), no studies to date have investigated its development across multiple academic semesters.

## 2 University instruction and public health measures during the COVID-19 pandemic in Germany

**Figure 1:**
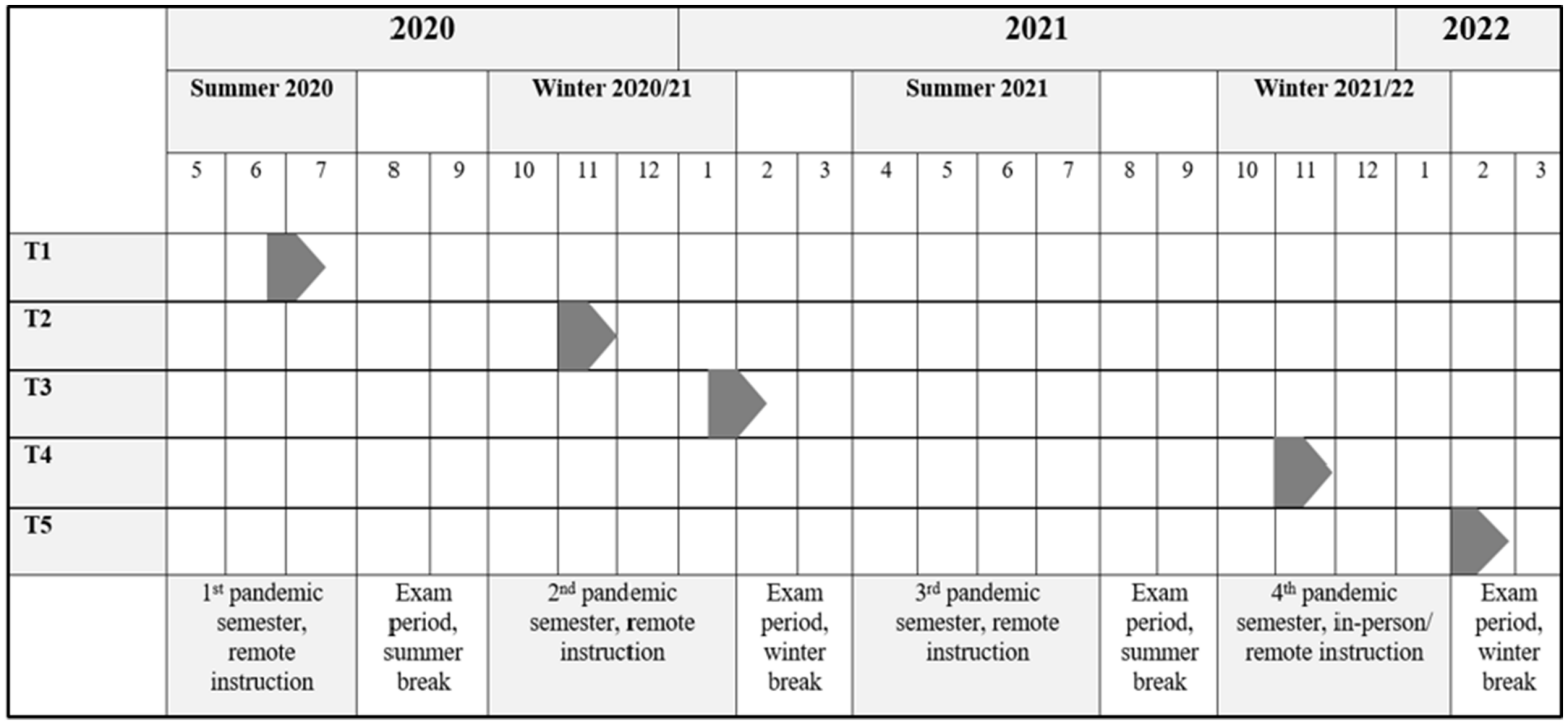
Timeline of data collection across five time points (T1–T5) during the COVID-19 pandemic (2020–2022). Information on the pandemic-specific situation in Germany during the study period: T1–T3 (remote instruction) – strict public health measures, including contact restrictions in regions with high COVID-19 case numbers, hygiene campaigns, and a nationwide lockdown (T3). T4 (in-person/remote instruction) – nationwide regulations to control infections, including restricted university access and a broad booster vaccination rollout. T5 (remote instruction) – restrictions due to the Omicron variant. For all details on the specific COVID-19 situation in Germany during the study period, see Supplementary Table 3. A similar version of Figure 1 was also published in Höhne et al. (2024).

In response to the high average daily number of new cases and in accordance with government and public health mandates, universities in Germany largely operated under remote instruction during the first year and a half of the pandemic (summer 2020 to winter 2021/22; in our study: T1–T3; see Bundesministerium für Gesundheit Deutschland, 2023). During this period, strict public health measures, including contact restrictions in regions with high COVID-19 case numbers, hygiene campaigns, and, in early 2021 (T3), a nationwide lockdown, were in place (see Bundesministerium für Gesundheit Deutschland, 2023). As a consequence, university students generally studied alone from home, with reduced interaction and fewer opportunities to build academic collaboration networks with other students compared to pre-pandemic times (e.g., Elmer et al., 2020). These conditions were associated with students experiencing study-related stressors in online learning environments, such as difficulties interacting with instructors and peers (e.g., Elmer et al., 2020; Hollister et al., 2022) and managing academic work (e.g., Matos Fialho et al., 2021). As health measures eased, in winter 2021/2022 (in our study: T4), universities had resumed in-person instruction, while nationwide regulations to control infections, including restricted university access and a broad booster vaccination rollout, shaped campus life (see Bundesministerium für Gesundheit Deutschland, 2023).^1^ In this context, despite the greater availability of interpersonal relationships on campus—including perceived support from instructors and peers (e.g., Cipolletta et al., 2025)—students nevertheless reported study-related stressors, such as social unease when speaking in front of groups (e.g., Karnbach et al., 2024). By early 2022 (in our study: T5), following restrictions due to the SARS-CoV-2 Omicron variant (see Bundesministerium für Gesundheit Deutschland, 2023), students reported study-related stressors such as the renewed closure of educational institutions (for high school students, see Neugebauer et al., 2024; for an overview of the period in our study, see Figure 1; for more information on prevalence rates, case numbers, tests, vaccine doses, as well as the general situation and public health measures in Germany, see Supplementary Table 3).

## 3 Thriving among university students and the potential impact of the pandemic

Despite considerable variation in the theoretical and methodological conceptualization of thriving (for an overview, see Brown et al., 2017), there is general agreement that thriving encompasses two core dimensions: vitality (affective) and learning (cognitive; Porath et al., 2012; Spreitzer et al., 2005; for a recent example, see Warnock et al., 2024). Individuals who thrive at work experience personal growth by feeling energized and by acquiring and applying knowledge, continually improving. Thriving is contingent on individual and social factors (e.g., support; Kleine et al., 2019) and can positively impact work-related outcomes such as health (e.g., Kleine et al., 2023), career satisfaction (e.g., Jiang et al., 2020), and performance (e.g., Christensen-Salem et al., 2021).

We chose to focus on the construct of thriving, as operationalized by Porath et al. (2012), for two main reasons. First, thriving offers a narrowly defined and theoretically coherent measure focused specifically on perceived psychological growth (Porath et al., 2012; Spreitzer et al., 2005), as opposed to broader, multidimensional constructs conceptually close to thriving, such as well-being or flourishing, which assess general psychological functioning and include psychological growth as just one of several dimensions (e.g., Diener et al., 2010; Keyes, 2002; Ryff, 1989; Ryff and Keyes, 1995). While thriving similarly captures both hedonic (i.e., vitality, focused on pleasure and happiness) and eudaimonic aspects (i.e., learning, focused on personal growth and self-realization; see Ryan and Deci, 2001; Ryff et al., 2021; Spreitzer et al., 2005), it excludes broader aspects such as social relationships, which are central to well-being and flourishing measures but can instead function as predictors rather than components of thriving (e.g., Brown et al., 2017). Second, as the construct of thriving and its measurement were explicitly developed and validated for use in workplace contexts (Porath et al., 2012), it provides a theoretically grounded measure that is likely also suitable for examining students’ thriving in higher education contexts (e.g., Haase et al., 2025; Ozcan et al., 2023; for other context-specific but broader measures of psychological functioning at work, see Rautenbach and Rothmann, 2017; Van Katwyk et al., 2000; for higher education contexts, see Renshaw and Bolognino, 2016; for school contexts, see Kern et al., 2015).

Thriving at work under challenging circumstances is considered a transient, dynamic psychological state that fluctuates within the same individual over time (e.g., Kleine et al., 2023). Research has shown that a substantial proportion of the total variance in vitality and learning over time is caused by within-person variations over small or moderate periods (i.e., over several weeks or over one to several months; e.g., Bensemmane et al., 2018; Brown et al., 2021; Kleine et al., 2023; for variations within a single day, see Niessen et al., 2012). These fluctuations can particularly arise during substantial changes and increased challenges in an individual’s work life. For example, Porath et al. (2012) found that the thriving levels of managers with significant responsibilities were higher 1 month after completing a leadership development training course than during the course. This difference was possibly due to contextual changes (i.e., transitions between work and private life settings) and work-related (interpersonal) changes (e.g., shifts in task structures and work roles), as well as to increased challenges. Analogously, in higher education, students were confronted with contextual changes (i.e., transitions between on campus and at-home studying) and work-related (interpersonal) changes (e.g., shifts in exam formats and difficulties in establishing social relationships during remote instruction) during the pandemic—as well as with increased challenges.

Cross-sectional studies found that university students reported moderate thriving levels during the height of the pandemic (in our study: T3 or shortly thereafter), when remote instruction was carried out (Sahin and Tuna, 2022; Warnock et al., 2024). The only longitudinal study on thriving among university students during the pandemic found that international students’ levels of thriving fluctuated daily over a 9-day period at the beginning of the pandemic, during remote instruction (Hua et al., 2022).

Longitudinal analyses of positive outcomes conceptually close to thriving during the first year of the pandemic (in our study: T1–T3) have shown decreases in students’ well-being toward fall 2020 (in our study: T2) and early 2021 (in our study: T3; e.g., Pasupathi et al., 2022; for overviews, see Buizza et al., 2022; Lemyre et al., 2023), and decreases in students’ study satisfaction toward early 2021 (in our study: T3; e.g., Gadosey et al., 2022). After students had returned to campus in fall 2021 (in our study: T4), cross-sectional studies showed that university students reported lower levels of well-being than pre-pandemic population estimates (e.g., Liverpool et al., 2023). To our knowledge, there are no longitudinal studies on university students’ positive outcomes during that period, only a longitudinal study on high school students that has shown decreases in students’ life satisfaction from early 2021 onward (in our study: T3–T5; Neugebauer et al., 2024).

With regard to the beginning of the pandemic during remote instruction (in our study: T1–T3), on the one hand, based on the aforementioned research on constructs conceptually close to thriving, it could be expected that students’ levels of thriving decreased toward fall 2020 (in our study: T2) and early 2021 (in our study: T3). On the other hand, because thriving is considered conceptually distinct from these positive outcomes (e.g., by encapsulating a specific learning dimension; for an overview, see Brown et al., 2017), and, as noted above, because positive outcomes such as thriving can emerge as responses to stressors when they are experienced as challenges (Feeney and Collins, 2015; Lazarus and Folkman, 1984), it is possible that thriving levels increased toward fall 2020 (in our study: T2) and early 2021 (in our study: T3). It is also possible that students had already developed greater resilience (i.e., maintaining their typical level of functioning despite ongoing stressors or by showing a homeostatic return to prior levels of functioning after adversity) during this period, such that thriving levels might not have changed (see Carver, 1998; O’Leary and Ickovics, 1995). With regard to the return to in-person instruction in fall 2021 (in our study: T4), compared to remote instruction at the beginning of 2021 (in our study: T3), which can be considered a substantial contextual and work-related (interpersonal) change within the period of our study, we expected students’ thriving levels to increase toward T4. We expected such increases, because, as already outlined, thriving is contingent on social factors in work contexts during face-to-face interactions (e.g., support from others; Brown et al., 2017; Frazier and Tupper, 2018; Imran et al., 2020; Kleine et al., 2019)—factors that had once again become more directly accessible to students. With regard to potential changes in thriving levels toward early 2022 (in our study: T5), when a large proportion of faculty members had returned to remote instruction, it could be expected that students’ thriving would decrease due to renewed changes, particularly characterized by the absence of social factors in face-to-face settings. This would also be in line with decreased levels of life satisfaction of high school students toward early 2022 (Neugebauer et al., 2024). However, it is also possible that students perceived the renewed closure of universities as a challenge, leading to increases in thriving levels (Lazarus and Folkman, 1984), or, that they had already developed greater resilience, resulting in no changes in thriving levels (see O’Leary and Ickovics, 1995).

## 4 Gender and college generation status as potential predictors of university students’ thriving over time

Pre-pandemic longitudinal evidence from work contexts has predominantly found no gender differences in thriving (e.g., Brown et al., 2021; Kaltenbrunner et al., 2019; Kleine et al., 2023). For example, Brown et al. (2021) have shown that sport performers’ thriving was predicted by their recent experiences of thriving, irrespective of gender. However, there are exceptions with studies on adolescents showing gender effects on thriving over time, such as girls’ levels of thriving fluctuating more than boys’ over a three-year period (e.g., Shek and Zhu, 2020).

While a large body of research has shown the detrimental effects of the pandemic on women’s mental health (e.g., anxiety, depression) and their stress experiences (e.g., Dotsikas et al., 2023; Gestsdottir et al., 2021; Prowse et al., 2021), longitudinal studies examining gender differences in positive psychological outcomes over the first year and a half of the pandemic remain scarce. Cross-sectional studies have shown that women reported lower levels of life satisfaction (e.g., Rogowska et al., 2021; Zoch et al., 2022) at the beginning of the pandemic (in our study: T1) and lower levels of well-being as the pandemic unfolded (e.g., Barbayannis et al., 2022; in our study: T2) than men. Regarding the period after the beginning of 2021 (in our study: T3–T5), studies have mostly shown declines in positive outcomes, irrespective of gender (for life satisfaction, see Neugebauer et al., 2024). With regard to the return to in-person instruction (in our study: T4), previous research has found that women thrive more than men during social interactions in face-to-face settings (Di Milia and Jiang, 2024). Specifically, the relationship between leader–member exchange (i.e., mutual affect, loyalty, perceived contribution to goals, and professional respect) and thriving was stronger for female than for male workers.^2^ This pattern may be related to women’s stronger focus on communion compared to men (e.g., Eagly, 1987; Kite et al., 2008; Kosakowska-Berezecka et al., 2023). Accordingly, it could be expected that female students would show greater increases in their thriving levels when social factors became more readily accessible again. However, research has also shown that men rated themselves as similarly communal as women (e.g., Obioma et al., 2022). Taken together, based on the mixed pre-pandemic and pandemic research, and, because, as already outlined, thriving is considered conceptually distinct from the aforementioned positive outcomes (see Brown et al., 2017), it remains unclear from existing research whether fluctuation differences in female and male students’ levels of thriving over the course of the pandemic could be expected.

To our knowledge, there is neither cross-sectional nor longitudinal research that has systematically examined college generation status as a predictor of thriving, operationalized as a joint sense of vitality and learning. In general, first-generation (FG) students face unique (study-related) stressors compared to continuing-generation (CG) students, such as a lack of familiarity with academic culture and financial barriers (e.g., Atherton, 2014). In line with this, pre-pandemic cross-sectional research has shown that FG students reported lower levels of constructs conceptually close to thriving, such as personal growth (Bowman, 2010), life satisfaction (Jenkins et al., 2013) or engagement (Pike and Kuh, 2005) than CG students. The pandemic seemed to exacerbate the already challenging study situation of FG students, who now faced specific (study-related) stressors, such as remote learning (including lack of reliable internet access), increased financial difficulties, and housing insecurities (e.g., Regan et al., 2023; Soria et al., 2020). Although this demanding situation was related to greater impairments in their mental health, alongside simultaneously lower rates of mental health service use compared to CG students at the beginning and during the height of the pandemic (e.g., Fruehwirth et al., 2021; Lipson et al., 2023; Regan et al., 2023; Soria et al., 2020; in our study: T1–T3), levels of flourishing, which is also conceptually close to thriving, did not differ between FG and CG students from the beginning of the pandemic until the end of the academic year in 2021 (Lipson et al., 2023; in our study: T1–T5). In summary, and based on the research discussed above, it does not seem clear how FG students responded to those specific (study-related) stressors in terms of thriving over the course of the pandemic compared to CG students.

## 5 The present research

Based on research showing that thriving levels can fluctuate over moderate periods of time, we were interested in potential variations in university students’ thriving levels over the course of two academic years during the pandemic, specifically from summer 2020 to the beginning of 2022 (in our study: T1–T5). While we explored most of the potential changes toward the time points (T1–T2, T2–T3, T4–T5), we predicted that students’ thriving levels would increase toward T4, when universities had returned to in-person instruction in fall 2021, compared to T3, when instruction was delivered remotely. Based on the mixed findings regarding gender as a predictor of thriving over time, and because from research it is not clear how college generation status would predict thriving, we explored changes toward all time points with regard to differences between female and male students, as well as between first-generation (FG) and continuing-generation (CG) students.

## 6 Materials and methods

### 6.1 Participants

We used data from a study on students’ experiences during the pandemic at the Faculty of Humanities of a large public university (more than 30,000 students) in Lower Saxony, Germany. Students were contacted twice per semester via a university mailing list and could join the study at any time point by providing their written consent. All administered surveys (T1–T5) are shown in Figure 1. Our overall sample consisted of 1,909 students who provided information on at least one of our variables of interest in the present study. Of these, 431 students completed at least two surveys and were included in our analyses (for additional study sample information by time point, see Supplementary Table 2). Our sample included all demographic groups of the faculty’s student population, with an overrepresentation of female students (78% female; 48% first-generation students).^3^

### 6.2 Measures

#### 6.2.1 Thriving

Students’ thriving was measured using a shortened and translated version of the Thriving at Work Scale by Porath et al. (2012). The scale consisted of three items (i.e., “I feel alive and vital,” “I feel energized,” “I am not moving forward”) using a 5-point Likert scale (1 = strongly disagree, 5 = strongly agree). Items 1 and 2 represent the vitality dimension of thriving, and item 3 represents the learning dimension (reverse-coded). All items showed good internal consistency (T1–T5: Cronbach’s *α* = 0.80–0.84).

#### 6.2.2 Demographics

Students indicated their gender (0 = female, 1 = male) and college generation status (0 = continuing-generation student, 1 = first-generation student) at the end of the survey.

### 6.3 Statistical analyses

If not stated otherwise, we analyzed our data using Mplus version 8.5 (Muthén and Muthén, 1998–2017). Prior to our main analyses, we examined missing data patterns in our sample using Little’s missing completely at random (MCAR) test (Little, 1988) within SPSS’s Missing Value Analysis option (version 28.0; IBM Corp, 2021). Additionally, we tested longitudinal measurement invariance (Liu et al., 2017).

Based on research on within-person fluctuations in thriving over moderate periods (e.g., Brown et al., 2021), we were interested in changes in thriving between immediately consecutive time points (e.g., T1–T2, T2–T3). To address this, in our main analysis, we used a neighbor change model, a subtype of latent change score (LCS) models (Geiser, 2010, 2013). We first specified a neighbor change model without predictors to assess model fit and examine the overall pattern of intraindividual change in students’ thriving over time (e.g., Ferrer and McArdle, 2010). Next, we simultaneously included gender and college generation status as predictors of students’ thriving at T1 and intraindividual changes over time, given that these variables may be interrelated through intersecting identities (e.g., Wright et al., 2023). Alternative model structures (e.g., latent autoregressive models; Geiser, 2010) were deemed less appropriate due to their emphasis on the relative interindividual stability of constructs over time and the indirect modeling of change through residuals. In contrast, LCS models directly capture change via latent difference variables (e.g., Geiser, 2010; Kleinke et al., 2017; McArdle and Hamagami, 2001; Steyer et al., 1997), aligning more closely with our interest in modeling dynamic intraindividual processes across specific consecutive time points. Missing values were estimated using full information maximum likelihood (FIML; Enders, 2010). Unlike traditional imputation methods, FIML directly estimates model parameters using all available data without imputing missing values and generally yields less biased and more efficient parameter estimates than other methods under both MCAR and MAR conditions (Enders and Bandalos, 2001; in our case: MCAR).

## 7 Results

### 7.1 Preliminary analyses

According to Little’s MCAR test, the data (χ2 = 227.40, df = 220, *p* = 0.352) were missing completely at random. Based on the criteria proposed by Chen (2007), longitudinal measurement invariance was supported, with full configural and metric invariance and partial scalar invariance across all five time points (for detailed model fit indices, see Supplementary Table 1).

### 7.2 Latent change score model results

**Figure 2:**
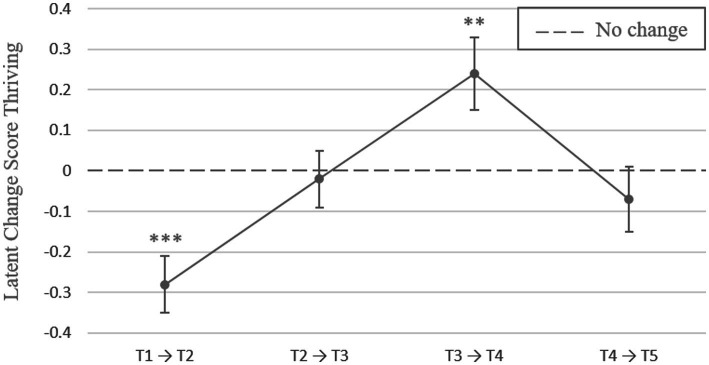
Latent change scores for students’ thriving across consecutive time points over the course of two academic years during the COVID-19 pandemic (2020–2022). Error bars represent standard errors. The dashed line indicates no change. Asterisks represent statistically significant changes (**p < 0.01; ***p < 0.001).

Students reported above-average levels of thriving at the beginning of the study period (T1, summer 2020; estimate = 3.01, *SE* = 0.06, *p* ≤ 0.001). From T1 toward T2 (fall 2020), during remote instruction, students had significant intraindividual decreases (estimate = −0.28, *SE* = 0.07, *p* ≤ 0.001). After these 6 months, from T2 toward T3 (early 2021), students’ thriving levels did not change when universities continued with remote instruction (estimate = −0.02, *SE* = 0.07, *p* = 0.833). As expected, we found intraindividual increases from T3 toward T4 (fall 2021), when universities had returned to in-person instruction (estimate = 0.24, *SE* = 0.09, *p* ≤ 0.01). There were no intraindividual changes from T4 toward T5 (early 2022), when universities had resumed remote instruction (estimate = −0.07, *SE* = 0.08, *p* = 0.381; for all results, see Table 1 and Figure 2; for the means of students’ thriving across all time points, see Supplementary Figure 1).

**Table 1:** Latent neighbor change score model of students’ thriving over the course of two academic years (2020–2022).

Time point/Change interval	T1		T1–T2		T2–T3		T3–T4		T4–T5	
	Estimate	(*SE*)	Estimate	(*SE*)	Estimate	(*SE*)	estimate	(*SE*)	estimate	(*SE*)
Intercept	**3.01**	**(0.06)**	**−0.28**	**(0.07)**	−0.02	(0.07)	**0.24**	**(0.09)**	−0.07	(0.08)

*Note*: *N* = 431. Model fit: RMSEA = 0.029, CFI = 0.977, TLI = 0.974, SRMR = 0.072. Bold font: *p* ≤ 0.05.

Neither students’ thriving at T1 nor their intraindividual changes in thriving toward later time points differed by demographic background—that is, by gender or college generation status (for all results, see Table 2).

**Table 2:** Latent neighbor change score model of students’ thriving over the course of two academic years (2020–2022) by gender and college generation status.

Time point/Change interval	T1		T1–T2		T2–T3		T3–T4		T4–T5	
	Estimate	(*SE*)	Estimate	(*SE*)	Estimate	(*SE*)	Estimate	(*SE*)	Estimate	(*SE*)
Intercept	**2.93**	**(0.14)**	−0.21	(0.16)	0.03	(0.15)	0.11	(0.13)	−0.23	(0.14)
Female	−0.07	(0.16)	−0.02	(0.18)	0.15	(0.19)	−0.20	(0.20)	0.25	(0.18)
CGS	0.14	(0.21)	−0.09	(0.24)	−0.15	(0.23)	−0.35	(0.18)	0.28	(0.15)
*R* ^2^	0.01		0.01		0.06		0.10		0.22	

*Note*: *N* = 425. Students who indicated a diverse gender identity (*n* = 6) were excluded. Gender (0 = female, 1 = male). College generation status (0 = continuing-generation students, CGS, 1 = first-generation students, FGS). Model fit: RMSEA = 0.031, CFI = 0.969, TLI = 0.961, SRMR = 0.078. Bold font: *p* ≤ 0.05.

## 8 Discussion

Understanding how university students psychologically adapt to profoundly challenging and disruptive times is essential for advancing theories of positive functioning. Thriving, defined as a joint experience of vitality and learning (Spreitzer et al., 2005), has attracted scholarly interest as a particularly positive outcome of adaptation to stressors. While it has been widely conceptualized as a dynamic state responsive to contextual and interpersonal factors (Kleine et al., 2023; Porath et al., 2012; Spreitzer et al., 2005), research has predominantly examined it in workplace settings, with comparatively less attention to how it fluctuates within individuals in educational contexts, particularly during periods of heightened contextual instability, such as the COVID-19 pandemic. The present study addresses this gap by being the first to longitudinally track intraindividual fluctuations in university students’ thriving across two academic years during the pandemic. Specifically, we examined the development of students’ thriving over five time points in Germany from summer 2020 to the beginning of 2022 (T1–T3: remote instruction; T4: in-person instruction/remote instruction; T5: remote instruction), periods marked by varying degrees of study-related individual, social, and instructional stressors. Complementing research on the role of gender in thriving, we further investigated whether the potential fluctuations differed for female and male students. In addition, our study adds to the literature by examining another characteristic of students’ demographic background that has not yet been examined in relation to thriving over time: students’ college generation status. We found intraindividual decreases of students’ levels of thriving toward fall 2020 during remote instruction and increases toward fall 2021 when universities had returned to in-person instruction. Intraindividual changes in thriving toward all later time points did not differ by gender or by college generation status.

### 8.1 University students’ thriving during remote instruction

While our data do not permit causal conclusions—given the limited scope of predictors included—we offer several theoretically grounded interpretations of the observed patterns.

At the onset of the pandemic (in our study: T1), university students reported slightly above-average thriving levels, aligning with cross-sectional study findings of moderate thriving levels among university students during the height of the pandemic (Sahin and Tuna, 2022; Warnock et al., 2024). Students showed intraindividual decreases in their experiences of thriving from the onset of the pandemic toward fall 2020 (in our study: T2). This pattern is consistent with declines observed in related constructs such as students’ well-being (e.g., Buizza et al., 2022) and study satisfaction (e.g., Gadosey et al., 2022) during the first academic year of the pandemic. Although not directly assessed in our study, the observed decline may reflect study-related stressors in online learning environments perceived as threatening (see Lazarus and Folkman, 1984), including reduced opportunities for interaction with instructors and peers (e.g., Elmer et al., 2020; Hollister et al., 2022) and stressors associated with transitioning academic work to virtual learning environments (e.g., El-Sakran et al., 2022). Despite evidence that students’ study satisfaction also decreased during winter 2020/2021 (Gadosey et al., 2022), we found no changes in students’ thriving from fall 2020 toward early 2021 (in our study: T3). One possible explanation is that students faced fewer disruptive changes in their academic life during this period. In contrast to the early stages of the pandemic, when digital tools and assessment formats were still less established, students in winter 2020/2021 may have begun to adapt, drawing on prior experience and developing learning- and performance-related (interpersonal) coping strategies (see Liebendörfer et al., 2023). This, in turn, may have resulted in greater continuity in students’ academic routines, and, consequently, stable levels of thriving (see Porath et al., 2012). Such a pattern aligns with frameworks describing distinct levels of functioning in response to adversity (O’Leary and Ickovics, 1995; see also Carver, 1998). When facing adversity, individuals can exhibit resilience by maintaining their typical, baseline level of functioning despite ongoing stressors or by showing a homeostatic return to prior levels of functioning after adversity. Supporting this, research indicates that stress levels of university students also remained stable during this period (e.g., Weber et al., 2022), despite escalating COVID-19 case numbers and renewed lockdown measures in Germany in winter 2020/2021 (Bundesministerium für Gesundheit Deutschland, 2023).

### 8.2 Contextual and interpersonal changes at university as enhancers of students’ thriving?

As expected, a key finding of our study was the pattern of intraindividual increases in students’ thriving levels toward fall 2021 (in our study: T4), when students had returned to their classrooms. This increase occurred despite ongoing study-related stressors such as social unease (Karnbach et al., 2024) and may be partly attributable to the renewed availability of in-person social interactions, which had previously been limited or absent during the first year and a half of remote instruction (e.g., Elmer et al., 2020; Hollister et al., 2022). In particular, supportive relationships with instructors and peers (see Imran et al., 2020; Kleine et al., 2019) may have offered key social coping resources that enabled students to successfully manage the study-related stressors upon returning to campus, thereby facilitating their thriving (see Feeney and Collins, 2015). This interpretation is consistent with a recent study on university students’ social belonging as a predictor of thriving during in-person instruction. Haase et al. (2025) found that the extent to which university students felt that they were part of their study program contributed to their experiences of vitality and learning. Social belonging is often positively related to the perceived availability of social support, for example, from instructors and peers at university (e.g., Walton and Cohen, 2007; see also Chiu et al., 2016). Moreover, research suggests that social belonging can be conceptualized as an indicator of the perceived quality of one’s interpersonal relationships in a given context (e.g., Shook and Clay, 2012; Walton and Cohen, 2007; see also Li et al., 2025). The return to campus may have increased the salience of the contextual environment (i.e., the respective study program), thereby strengthening students’ sense of social belonging and contributing to their thriving levels. However, these considerations remain speculative, as our study could not include social predictors of thriving across time due to the limited sample size and therefore cannot establish direct links between social experiences and thriving.

### 8.3 Students’ thriving over time: irrespective of gender and college-generation status

Consistent with pre-pandemic longitudinal research (e.g., Brown et al., 2021; Kaltenbrunner et al., 2019; Kleine et al., 2023), intraindividual changes in students’ thriving toward all later time points did not differ by gender. While not empirically tested in our study, it seems that both female and male students lacked sufficient coping resources to sustain thriving during the unprecedented study situation at the beginning of the pandemic (see Lazarus and Folkman, 1984), which likely contributed to similar decreases toward fall 2020 (in our study: T2). Interestingly, studies have found greater mental health impairments—such as higher stress levels and lower levels of well-being—among female compared to male students during this period (e.g., Barbayannis et al., 2022; Gestsdottir et al., 2021; Prowse et al., 2021). This may indicate that thriving in higher education reflects a relatively broad form of adaptive psychological functioning that is less sensitive to gender differences than other indicators, such as well-being or stress, which are more closely tied to immediate emotional states (see Graves et al., 2021; Ryff et al., 2021). In general, psychological functioning and development can include hedonic and eudaimonic dimensions (e.g., Ryan and Deci, 2001; Ryff et al., 2021), as already outlined. Since thriving has been proposed to encompass both hedonic (vitality) and eudaimonic (learning) dimensions (Spreitzer et al., 2005), it can be suggested that the interplay of these dimensions in thriving involves more stable aspects of personal development, which are suggested to be less sensitive to gender differences than hedonic well-being, which consists of less stable states of positive emotions (see Graves et al., 2021; Ryff et al., 2021). This may also explain the comparable patterns of stability in thriving levels observed among both female and male students beyond the first six months (in our study: T2–T3).

Following the return to in-person instruction (in our study: T4), both female and male students’ thriving levels increased. This similar pattern of change is notable given that prior research suggests that thriving through social factors may be related to gender role beliefs (Feeney and Collins, 2015), with women often (self-)described as more community-oriented than men (e.g., Eagly, 1987; Kite et al., 2008; Kosakowska-Berezecka et al., 2023). Supporting this, women have been shown to thrive more during social interactions in face-to-face settings. For example, the association between positive leader–member exchange (e.g., mutual affect, professional respect) and thriving has been found to be stronger for women than for men (Di Milia and Jiang, 2024). However, first evidence suggests that men perceive themselves as similarly communal as women (e.g., Obioma et al., 2022), which could help explain why both genders experienced similar increases in their thriving levels when social coping resources became more accessible. Nonetheless, our study does not identify the specific social (or individual) coping resources that may have contributed to these gains in thriving among female and male students after the return to in-person instruction.

Likewise, first-generation (FG) and continuing-generation (CG) students showed similar developments in their thriving. This aligns with research showing that levels of flourishing did not differ between FG and CG students from the beginning of the pandemic until the end of the academic year in 2021 (Lipson et al., 2023; in our study: T1–T5)—despite the greater mental health impairments typically reported by FG students during the first academic year of the pandemic (e.g., Fruehwirth et al., 2021; Soria et al., 2020). Although not directly assessed, one possible explanation for the similar decreases toward fall 2020 (in our study: T2) and the similar increases in FG students’ thriving after having returned to campus (in our study: T4), compared to CG students, may be off-campus interpersonal relationships, social coping resources FG students frequently draw on. For instance, in a qualitative study, associations were found between FG students’ well-being and their off-campus interpersonal relationships in their home communities, in addition to on-campus social interactions (McCarron, 2022). These off-campus interpersonal relationships may have buffered against stronger decreases and supported similar increases in thriving levels compared to CG students.

### 8.4 Limitations and future directions

First, although our study adds to the literature on positive outcomes as responses to the specific study situation during the pandemic, we could not include pre- and post-pandemic time points and therefore are not able to separate the specific effects of the pandemic from general effects during university students’ studies. For example, research has shown that positive psychological states generally tend to decrease over the course of students’ studies (for well-being, see Bewick et al., 2010; Cobo-Rendón et al., 2020).

Second, another limitation pertains to the time period examined in our study. Based on research that has investigated thriving over moderate periods in work contexts (Brown et al., 2021; Kleine et al., 2023), it seemed crucial to investigate university students’ thriving over a longer period. However, to gain a deeper understanding of the microdynamics of thriving in higher education, future research should examine university students’ thriving over shorter periods using methods such as experience sampling or diary studies (e.g., Hua et al., 2022; Niessen et al., 2012).

Third, our study did not include social predictors of thriving, limiting our ability to test whether changes in the availability of social coping resources actually contributed to the observed fluctuations. An interesting next step would be to examine how social coping resources—such as perceived support from instructors and peers—relate to students’ thriving over short and moderate time periods (for the role of host country support in international students’ thriving over a 9-day period, see Hua et al., 2022). In addition, future studies could investigate the underlying mechanisms through which social factors, such as close relationships, contribute to long-term thriving in higher education (Feeney and Collins, 2015). For example, in workplace contexts, a recent longitudinal study identified organization-based self-esteem as a mediator between social coping resources and thriving (Kim and Beehr, 2022). In developing such models, gender should be systematically considered, as it may shape the extent to which students benefit from social support. Although both female and male students showed increased levels of thriving after the return to in-person instruction, these patterns should be replicated in higher education, given that gender role beliefs—unmeasured in our study—may moderate how social coping resources are experienced and utilized (Feeney and Collins, 2015; Kosakowska-Berezecka et al., 2023).

Fourth, the lack of significant effects for gender and college generation status should be interpreted with caution due to the sample composition and missing data. The unequal gender distribution, with a smaller number of male participants, may have reduced the statistical power to detect gender-related effects (Aguinis, 1995; Alexander and DeShon, 1994). However, by accounting for measurement error through the use of latent variables, LCS models can enhance the overall precision of parameter estimates (e.g., Kline, 2016). While missing data on college generation status were handled via full information maximum likelihood (FIML; e.g., Lee and Shi, 2021; Newman, 2014), this approach does not fully mitigate the risk of reduced estimate precision and increased standard errors that can result from missing data.

Fifth, we used a shortened three-item version of the Thriving at Work Scale by Porath et al. (2012) to ensure longitudinal measurement invariance. While short scales can reduce conceptual breadth, reliability, and content validity (Credé et al., 2012; Marsh et al., 1998; Xiao et al., 2024), research shows that well-selected short scales can retain psychometric strength, often comparable to longer item versions (e.g., Gogol et al., 2014; Kjell and Diener, 2021; Kleine et al., 2023). Our items reflected both theoretical core dimensions of thriving, i.e., vitality and learning, suggesting that intraindividual changes in this three-item score still meaningfully captured changes in students’ overall thriving (see Porath et al., 2012). Still, future studies should replicate these findings using more comprehensive measures.

Lastly, because our study was limited to one specific university in one German state, our findings should not be generalized to the experiences of vitality and learning among university students in Germany during the pandemic. For instance, prevalence in Lower Saxony during our survey period was below the national average at most time points (see Supplementary Table 3), potentially leading to an overestimation of thriving increases at T4. However, particularly during earlier phases (T1–T3), infection rates were comparable to national levels.

### 8.5 Practical implications

Although our study focused on the unique circumstances of the COVID-19 pandemic, our findings may reflect more general patterns that are relevant to higher education institutions beyond this specific context. Accordingly, we outline several implications that may inform how higher education institutions can support students’ thriving across different stages of their studies.

Our findings show that students’ thriving can fluctuate substantially over two academic years (see Brown et al., 2021; Kleine et al., 2023). Given the dynamic nature of thriving, higher education institutions may benefit from systematically monitoring medium-term developments in students’ vitality and learning across academic terms. To implement such monitoring, short, validated thriving assessments (e.g., Porath et al., 2012; see also Haase et al., 2025) could be embedded into existing structures, such as institutional surveys on students’ mental health and well-being, administered in paper-and-pencil or digital formats (e.g., Lahtinen et al., 2023).

Given that fluctuations in thriving can occur in response to substantial changes (e.g., Porath et al., 2012; see also Brown et al., 2021), monitoring may help to examine whether changes in students’ thriving are associated with transitions between different study phases, for example, transitions from regular coursework to thesis work or high-stakes group projects. Given the important role of social relationships in fostering thriving (Feeney and Collins, 2015; Imran et al., 2020; Jiang et al., 2020), it seems important to examine how the relevance of various dimensions of social relationships with instructors and peers might shift across these transitions. Thriving could therefore be examined alongside instructor-related and peer-related factors that have been shown to support thriving in work contexts. Instructor-related factors encompass mentoring (see Jiang et al., 2020)—including challenging assignments and emotional support—and feedback on important academic decisions (see Kim and Beehr, 2020), such as students’ grades and academic progress (for a measure of instructors’ self-perceived ability to support students, see also Haase and Zander, 2022). After the transition from coursework to thesis work, for instance, students may particularly rely on individualized mentoring and expert feedback (see Jiang et al., 2020; Kim and Beehr, 2020), which may positively contribute to their thriving. At the peer level, relevant factors include support from fellow students (see Zhai et al., 2020), feelings of connection (see Carmeli and Spreitzer, 2009), and mutual recognition of strengths (see Moore et al., 2022). After the transition to demanding group-based tasks, for example, thriving may be particularly shaped by interpersonal dynamics such as feelings of connectedness (Carmeli and Spreitzer, 2009). Taken together, fluctuations in students’ thriving may partly reflect variation in the availability and quality of social relationships, resulting in greater intra- and interindividual variability (Brown et al., 2021; Kleine et al., 2023).

In light of our findings suggesting that remote instruction may pose specific challenges for students’ thriving, higher education institutions may pay particular attention to creating positive and socially supportive online learning environments, to, in turn, promote students’ thriving. Effective instructional strategies may include interactive communication tools that support direct exchange between instructors and students. These tools have been shown to increase students’ satisfaction—a construct conceptually close to thriving—through instructors’ social presence (i.e., students’ perception that instructors are attentive and involved in the online interaction; see Park and Kim, 2020). Equally important, collaboration tools that facilitate peer-to-peer interaction (see Maxrath et al., 2025), such as shared social annotation platforms, can enhance students’ perceived social belonging in online courses (Kelly and Clinton-Lisell, 2025). Social belonging, in turn, has been found to predict students’ thriving (see Haase et al., 2025).

As our findings suggest that thriving fluctuated not only across time but also appeared sensitive to shifts in the broader learning environment, targeted, phase-specific interventions may help buffer against declines and foster recovery (for a systematic review on peer support interventions in higher education, see Pointon-Haas et al., 2024).

## 9 Conclusion

Our findings offer initial evidence that university students’ thriving can fluctuate in response to study-related stressors and broader contextual shifts within the higher education environment. In particular, our results suggest that online learning environments may be less conducive to sustaining students’ vitality and learning, whereas in-person social interactions seem to play a facilitative role in supporting students’ thriving. We hope our findings underscore the potential of higher education institutions as developmental contexts in which students can thrive through challenges, connection, and change—a process that may, in turn, foster key outcomes such as performance. This potential for thriving may represent a particular added value of on-site universities compared to exclusively online learning environments.

## Data Availability

The raw data supporting the conclusions of this article will be made available by the authors, without undue reservation.

## Footnotes

^1^At the end of the winter term 2021/2022 (early 2022), many universities resumed remote instruction for a few months due to the SARS-CoV-2 Omicron variant.

^2^The findings from that study should be interpreted with caution due to the highly unbalanced gender distribution (95% female).

^3^Since college generation status was not assessed before T4, the longitudinal dataset contains missing values for this variable from T1 to T3.
