# Supplementary material for: University students’ thriving during remote and in-person instruction in the COVID-19 pandemic: longitudinal evidence from two academic years
Source: Front Psychol. 2026 Jan 6;16:1638392. doi: 10.3389/fpsyg.2025.1638392 (PMC12815859; doi:10.3389/fpsyg.2025.1638392)
Supplement: Supplementary file 1 [file Data_Sheet_1.pdf]

Supplementary Material

1 Supplementary Tables

1.1 Supplementary Table 1

Measurement invariance testing across time

Model	χ^2	df	*p*	RMSEA	CFI	SRMR
Configural	71.887	69	0.3825	0.010	0.998	0.050
Metric	93.519	85	0.2471	0.015	0.994	0.061
Scalar	106.383	92	0.1450	0.019	0.990	0.062

Note. Configural invariance: factor loadings and intercepts are not set equal across five time points. Metric invariance: factor loadings are set equal across five time points. Scalar invariance: factor loadings and intercepts are set equal across five time points. Based on the criteria by Chen (2007), we assumed measurement invariance when models did not differ by more than 0.010 in CFI, supplemented by 0.015 in RMSEA or 0.030 in SRMR. Partial scalar invariance was given with a freely estimated intercept of item 3 at T5.

1.2 Supplementary Table 2

Additional study sample information by time point

	T1	T2	T3	T4	T5
n	258	198	236	183	184
Female (*n*)	206 (80%)	155 (78%)	190 (81%)	140 (77%)	139 (76%)
CGS (*n*)	-	-	-	93 (51%)	98 (53%)

Note. *N* = 431 (i.e., students who completed at least two surveys and were included in our analyses). CGS = continuing-generation students. College generation status was not assessed before T4. Valid percentages of demographic data are reported.

1.3 Supplementary Table 3[1]

Relevant information on the COVID-19 pandemic by time point

Time point	Average daily new COVID-19 cases (per 100,000); average prevalence (per 100,000)		Average weekly tests administered	Average daily COVID-19 vaccine doses administered	Information on the general situation and public health measures
	Lower Saxony	Germany	Germany	Germany	Germany
T1 (June/July 2020)	24.88 (0.31); 13,613 (170.09)	401.17 (0.48); 196,053 (235.75)	510,896	0	Strict health measures in regions with particularly high incidence (*hotspot strategy*); testing strategy for people entering from abroad; additional funding for digital teaching services; expansion of recommended individual hygiene measures (e.g., regular ventilation and hand washing) and the use of the COVID-19 warning app
T2 (November 2020)	1,227 (15.34); 59,518 (743.66)	18,795 (22.60); 860,203 (1,034)	1,416,239	0	No significant changes compared to T1
T3 (January/ February 2021)	880.48 (10.97); 144,103 (1,795)	10,124 (12.16); 2,223,862 (2,672)	1,109,834	111,361	Lockdown (e.g., non-essential stores, daycare centers, cultural, catering, and service establishments closed; home office obligation; obligation to wear medical masks in enclosed public places; strict contact restrictions); vaccination campaign with first prioritization of targeted groups at high risk of severe COVID-19 progression (e.g., persons aged ≥ 80 or people exposed to a high risk such as individuals in medical professions)

[1] A similar version of Supplementary Table 3 was also published in Höhne et al. (2024).

T4 (November 2021)	1,768 (22.03); 339,441 (4,229)	41,714 (50.11); 5,016,139 (6,026)	1,569,243	305,644	Mandatory nationwide infection control measures, including the *3G rule* (vaccinated, tested, or recovered) for all persons meeting in publicly accessible indoor areas such as restaurants; a particularly large number of people received their third vaccination
T5 (February 2022)	15,611 (191.76); 761,462 (9,354)	197,435 (234.04); 11,336,518 (13,438)	2,555,112	225,520	Spread of the SARS-CoV-2 Omicron variant

Note. Data is based on the exact survey period of each time point. Daily new cases refer to the average number of cases on each day in relation to the number of days in the respective time period. The prevalence is the average total number of cases during the respective time period (new cases and all cases from the beginning of the COVID-19 pandemic). For comparison reasons between Lower Saxony and Germany, we additionally calculated the average daily new cases and the prevalence per 100,000 inhabitants. The population figures for the respective calendar year served as the basis for this calculation: Lower Saxony: 8,003,421 (2020), 8,027,031 (2021), and 8,140,242 (2022); Germany: 83,160,000 (2020), 83,240,000 (2021), and 84,360,000 (2022). Weekly tests administered refer to PCR tests only. Daily vaccine doses administered include all types of doses (i.e., first and second vaccinations, boosters). Health measures that were applied at the country level (Germany) generally corresponded to those at the federal state level (Lower Saxony). Values in the tens and hundreds range are rounded to two decimal places.

2 Supplementary Figures

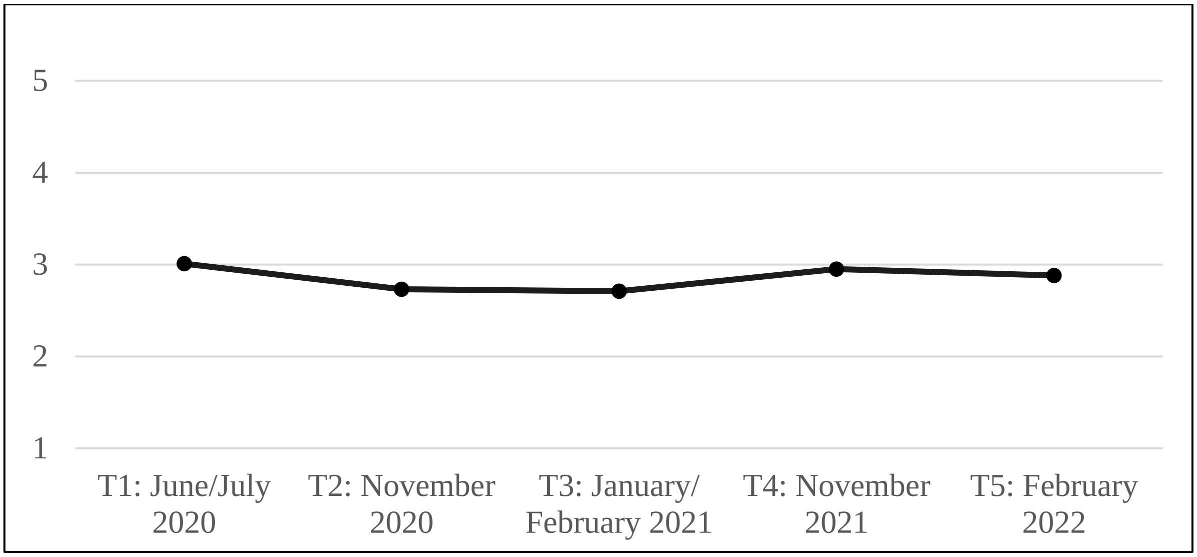

Supplementary Figure 1. Development of students' thriving across five time points (T1–T5) during the COVID-19 pandemic.

3 Supplementary References

Bundesministerium für Gesundheit Deutschland. (2023). *Coronavirus-Pandemie: Was geschah wann? Chronik aller Entwicklungen im Kampf gegen COVID-19 (Coronavirus SARS-CoV-2) und der dazugehörigen Maßnahmen des Bundesgesundheitsministeriums.* Available online at: https://www.bundesgesundheitsministerium.de/coronavirus/chronik-coronavirus.html (Accessed May 25, 2025).

Corona-zahlen-heute. (n.d.). *Aktuelle Corona Inzidenz von Niedersachsen*. Retrieved May 25, 2025, from https://corona-zahlen-heute.de/deutschland/niedersachsen/.

Corona-zahlen-heute. (n.d.). *Aktuelle Corona Inzidenz von Deutschland*. Retrieved May 25, 2025, from https://corona-zahlen-heute.de/deutschland/.

Chen, F. F. (2007). Sensitivity of goodness of fit indexes to lack of measurement invariance. *Struct. Equ. Model*. 14, 464–504. doi: 10.1080/10705510701301834

Höhne, E., von Keyserlingk, L., Haase, J., Arum, R., and Zander, L. (2024). Levels and facets of university students' stress during the COVID-19 pandemic: longitudinal evidence from the first two academic years in Germany and the U.S. *Soc. Personal. Psychol. Compass* 18:e12935. doi: 10.1111/spc3.12935

Our World in Data. (n.d.). *Daily COVID-19 vaccine doses administered*. Retrieved May 25, 2025, from https://ourworldindata.org/explorers/coronavirus-data-explorer?zoomToSelection=true&time=2020-03-01..latest&facet=none&country=~DEU&pickerSort=asc&pickerMetric=location&Metric=Vaccine+doses&Interval=New+per+day&Relative+to+Population=false&Color+by+test+positivity=false.

Robert Koch Institut. (2023). *Tabellen zu Testzahlen, Testkapazitäten und Probenrückstau pro Woche*. Available online at: https://www.rki.de/DE/Themen/Infektionskrankheiten/Infektionskrankheiten-A-Z/C/COVID-19-Pandemie/Testzahl.html (Accessed May 25, 2025).

Statistisches Bundesamt. (n.d.). *Bevölkerungsstand: Amtliche Einwohnerzahl Deutschlands 2022.* Retrieved May 25, 2025, from https://www.destatis.de/DE/Themen/Gesellschaft-Umwelt/Bevoelkerung/Bevoelkerungsstand/_inhalt.html.

Statistisches Bundesamt. (2023). *Einwohnerzahl in Niedersachsen von 1960 bis 2022*. Available online at: https://de.statista.com/statistik/daten/studie/155154/umfrage/entwicklung-der-bevoelkerung-von-niedersachsen-seit-1961/ (Accessed May 25, 2025).
